# Physiological Mechanism of Internode Bending Growth After the Excision of Shoot Sheath in *Fargesia yunnanensis* and Its Implications for Understanding the Rapid Growth of Bamboos

**DOI:** 10.3389/fpls.2020.00418

**Published:** 2020-04-23

**Authors:** Shuguang Wang, Hui Zhan, Pengcheng Li, Caihua Chu, Juan Li, Changming Wang

**Affiliations:** Key Laboratory for Sympodial Bamboo Research, Faculty of Life Sciences, Southwest Forestry University, Kunming, China

**Keywords:** bamboo shoot sheath, internode elongation, bending growth, water pressure, sugar metabolism

## Abstract

The physiological function of bamboo shoot sheaths is still unclear. In the present study, we investigated the anatomical and physiological influences of bamboo shoot sheaths on internode elongation by longitudinally striping parts of sheaths. The internodes would bend toward the bare sides during night. The results showed that amounts of water leaked at the cut of shoot sheaths during night, which impeded the increase of water, water pressure and assimilate transport rates, and decreased starch and soluble sugar catabolism in the bare side of the internodes. A higher level of water pressure and sugar metabolism increased the vacuole expansion and promoted the cell expansion in the outer sides as compared to the bare sides. The bending growth of internodes was mainly due to the significant differences in cell expansion, which was led by the difference in water pressure and sugar hydrolysis levels between the inner and outer sides. Bamboo internode elongation mainly relied on the increase of water pressure and soluble sugar concentration. Shoot sheaths played an important role in the rapid growth of bamboo shoots as a controller in water and assimilate transportation. This study gave a new insight into understanding the rapid growth mechanism of bamboo plants.

## Introduction

Bamboo plants are the fastest growing plants worldwide, and their culms can complete their height growth in one growing season (Panda, 2011). The mechanism underlying the rapid growth of bamboo shoots is a hot research topic. However, most works have focused on the molecular regulatory mechanism in order to identify the related genes (Peng et al., 2013; Yeh et al., 2013). The anatomical and physiological characteristics of young shoots during their rapid growth have been neglected.

Bamboo shoot sheaths are one important morphological characteristic in systematic bamboo classification (Janzen, 1976). They are green or some other color but are usually persistent to a certain degree even after turning brown, and enclose the bamboo internodes for some time (Wong, 2004). Most studies have focused on their mechanical functions, but few on the physiological functions. In the earliest stages of bamboo growth, the shoot sheath completely surrounds and protects new shoots (Singh et al., 2013). Wong (2004) also considered that shoot sheaths play an important role in protecting the tender lower part of a bamboo internode. Other studies showed that sheaths could avoid possible damage by providing crucial stiffness (Niklas, 1990, 1998; Zebrowski, 1992).

Unlike bamboo leaves, shoot sheaths have large areas of abaxial and adaxial surfaces and directly connect to the nodes of shoot culms via vascular bundles (Liese, 1998; Wang et al., 2018). As a unique organ of bamboo shoots, no other works or papers have ever involved their physiological functions on bamboo shoot elongation. Not yet fully understanding their specific physiological functions, we hypothesized that culm sheaths might play an important role during the shoot elongation process. Therefore, we decided to design a series of experiments to test the hypothesis and confirm the physiological functions of shoot sheaths in the rapid growth of bamboo shoots, including the effects of transverse and longitudinal cutting of bamboo shoot sheaths on shoot elongation. We have reported that culm sheaths and sheath blades were the main transpiration and respiration organs for bamboo shoots and young elongating culms; their transpiration rate and the stomatal conductance of culm sheaths were significantly higher than those of foliage leaf blades (Wang et al., 2018), but their physiological functions are still not very clear. It is very difficult to investigate their physiological functions as these sheaths are attached to the elongating bamboo shoots. Hence, we decided to cut the shoot sheaths in order to analyze the effects of their excision on bamboo internode elongation and to investigate their potential physiological functions during rapid shoot growth, in order to reveal their significance for rapid shoot growth.

In our previous studies, we have noticed that the elongation speed of young shoots decreased after the culm sheaths were cut off (Wang et al., 2018). When one strip of culm sheaths was peeled off longitudinally from the young culms, the culms would bend rapidly toward the bare sides in one night. However, few other related articles could systematically explain these phenomena or have reported similar results. Only Shang et al. (2013) have ever reported that they dwarfed the young culms of moso bamboos (*Phyllostachys edulis*) by stripping the culm sheaths, but they did not further analyze the relations between the rapid growth of bamboo shoots and the attached culm sheaths. The excision of shoot sheaths impeded the rapid growth of bamboo shoots or caused their bending growth, which suggested that shoot sheaths were intensively related to the rapid growth of bamboo shoots. The anatomical and physiological mechanism of shoot sheaths affecting the rapid elongation of bamboo internodes is still unclear. The influence of shoot sheaths on the nutrition transport and metabolism in rapidly elongating shoots is also unknown.

The growth of tissues can be explained by cell division and cell expansion, with cell expansion being strongly correlated to the water pressure and sugar metabolism in cells (Munns et al., 2000; Geitmann and Ortega, 2009; Hamant and Traas, 2010; Robinson et al., 2013; Beauzamy et al., 2014). Winter and Huber (2000) considered that invertase was associated with cell expansion, whereas Sucrose synthase (SUSY)-catalyzed metabolism was linked with biosynthetic processes (e.g., cell wall or storage products). Soluble acid invertase (SAI) activity was usually higher in rapidly growing tissues, such as cell and tissue cultures, root apices and immature stem internodes (Zhu et al., 1997). SUSY was also reported to play a role in the synthesis of fibers (Ruan et al., 1997). Invertase and SUSY were involved in the accumulation of soluble sugars and then higher soluble sugar contents acted as osmotica and further promoted cell expansion and growth (Yamada et al., 2009a, b). Magel et al. (2005) also considered that invertases were involved in the cell elongation and expansion of bamboo culms, possibly by modulating osmotic pressure and cell turgor. Additionally, the constant soluble sugar influx might also affect the turgor pressure of cells and their cell expansion. Therefore, the assimilate transport also needs to be investigated when bamboo internode elongation is considered.

The differences in cell expansion might lead to differences in anatomical characteristics between the bare culm walls and the walls covered with normal sheaths, including cell size and vascular bundle area. Hence, we hypothesized that the bending growth of bamboo internodes should be caused by differences in water and sugar influx, sugar metabolism, soluble sugar concentrations, as well as other factors, causing anatomical differences between the two sides of the bending internodes.

In the present study, we chose the young elongating *Fargesia yunnanensis* culms as study objects to analyze the physiological mechanism of the bending growth of bamboo internodes after the longitudinal excision of shoot sheaths, and further to probe the influences of shoot sheaths on water and sugar transport, and metabolism in the elongating shoot culms. This will boost our understanding of the rapid growth mechanism of bamboo shoots and provide new insights by analyzing the physiological functions of shoot sheaths in bamboo internode elongation.

## Materials and Methods

**FIGURE 1:**
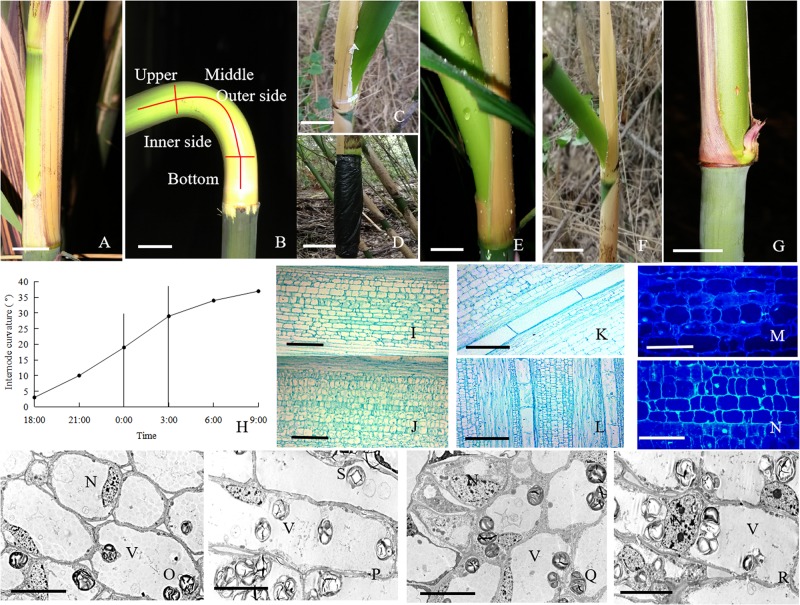
The morphological and anatomical observations on the bending internodes. **(A)** The sheath was peeled off at 17:00 p.m. Bar = 3 cm. **(B)** The bending internode was divided into the inner and outer sides in transverse direction and the upper, middle and bottom parts in longitudinal directions. Bar = 3 cm. **(C)** The internode bent slightly after 10 h during the diurnal time, when a strip of culm sheath was peeled off at morning. Bar = 3 cm. **(D)** After a strip of shoot sheath was peeled off at 17:00 p.m., the internode was covered with a black plastic film, in order to decrease the effects of light and temperature on the internode elongation. Bar = 4 cm. **(E)** The internode bent quickly at midnight (0:00 a.m.). Bar = 4 cm. **(F)** The completely bent internode at the next morning (9:00 a.m.). Bar = 2.5 cm. **(G)** The internode did not bend any more as its elongation completed. Bar = 3 cm. **(H)** The curvature degree of internodes at different times, showing the rapidly bending period from 24:00 p.m. to 3:00 a.m. **(I)** The parenchyma cells in the outer side of the bending internodes, showing longer size. Bar = 200 μm. **(J)** The parenchyma cells in the inner side of the bending internodes, showing shorter size. Bar = 200 μm. **(K)** The metaxylem vessel in the outer side of the bending internodes, showing longer size. Bar = 200 μm. **(L)** The metaxylem vessel in the inner side of the bending internodes, showing shorter size. Bar = 200 μm. **(M)** The nuclei of most parenchyma cells were localized close to the walls in the outer side of the bending internodes. Bar = 50 μm. **(N)** The nuclei were still observed in the center of parenchyma cells in the inner side of the bending internodes (arrow). Bar = 50 μm. **(O,P)** The transverse **(O)** and longitudinal **(P)** sections of the outer side of the bending internodes under TEM, showing bigger vacuoles, thinner cytoplasm layers and smaller nuclei. N, nucleus. S, starch grains. V, vacuole. Bar = 10 μm. **(Q,R)** The transverse **(Q)** and longitudinal **(R)** sections of the inner side of the bending internodes under TEM, showing smaller vacuoles, wider cytoplasm layers and bigger nuclei. N, nucleus; S, starch grains; V, vacuole. Bar = 10 μm.

*Fargesia yunnanensis* Hsueh et Yi is an alpine bamboo species, which is mainly distributed in the Sichuan and Yunnan Provinces of China. Its mature culms usually contain 29–30 internodes. The 8th internodes were chosen because they represent one of the longest internodes, reaching up to be 40 cm in length according to our field observations. The 20 cm long internodes of the 8th internodes were at the rapidly elongating stage, and about two-thirds of these internodes are enveloped by shoot sheaths. To observe the influences of culm sheaths on internode elongation, a strip (1 cm wide) of the sheaths surrounding the rapidly elongating internodes was longitudinally torn off from the top of the sheath to the sheath base in 10 rapidly elongating shoots (Figure 1A). The excision of shoot sheaths was conducted at 9:00 a.m., and the results were recorded at 17:00 p.m. The same experiments were repeated at 17:00 p.m. and the results were recorded at 9:00 a.m. the next day. The bending angles were consecutively recorded every 3 h from 18:00 p.m. to 9:00 a.m. The bending growth data was recorded and processed by using Microsoft Office 2010 (Microsoft Corporation, Redmond, WA, United States).

In order to analyze the dynamic changes of sugar metabolism in the bending internodes, samples were obtained from the bending internodes at midnight (24:00 p.m.) and on the next morning (9:00 a.m.) separately, i.e., 7 and 16 h after the strip of shoot sheaths was removed. As a control, samples were also obtained from the normal elongating internodes at the same time. All the bending internodes were divided into different parts using a sharp razor and taken back into the lab immediately. For determining the soluble sugars and starch contents and the activities of sucrose and starch metabolizing enzymes, the samples were stored in liquid nitrogen. For the anatomical observations, the samples were fixed in formalin-acetic acid-alcohol (FAA) fixative (1.85% formaldehyde, 45% alcohol, and 0.25% acetic acid).

### Anatomical Analysis

After a fixative in FAA solution, the specimens were cut into 7 μm thick paraffin sections by using a rotary microtome (Leica), and then were stained with safranin and fast green, in order to analyze the size differences of parenchyma cells, metaxylem vessels and vascular bundles between the inner and outer sides of the bending internodes.

In order to observe the distribution of the nuclei of bending internodes, the paraffin sections were dewaxed and rehydrated, and then were stained with Hoechst 33342 (2′-[4-ethoxyphenyl]-5- [4-methyl-1-piperazinyl]-2,5′-bi-1H-benzimidazole trihydrochloride trihydrate), which is a cell permeable DNA stain that can be excited by ultraviolet light and emit blue fluorescence at 460–490 nm.

The ultrathin sections were obtained using a diamond knife, and stained in 2% uranyl acetate for 15 min and lead citrate for 10 min. The ultrathin sections were examined and photographed under a JEM-1400 Transmission Electron Microscope (TEM), in order to observe the vacuolization of parenchyma cells in the inner and outer sides of the curved internodes.

The starch grains in the rapidly elongating internodes of *F. yunnanensis* were localized using the method of periodic acid-Schiff (PAS) reaction. The paraffin sections (7 μm) were soaked in 0.5% KIO_4_ for 10 min, followed by 30 min in Schiff’s reagent (Chu, 1963), and then were dehydrated in a graded series of ethanol and stained with Fast green FCF (Ameresco 0689, Solarbio, Beijing, China) (Fast green 1 g + clove oil 100 ml + 100% ethanol 100 ml). The sections were observed under a fluorescence microscope (Nikon E400, Tokyo, Japan).

### Endogenous Soluble Sugars and Starch Contents Determination

The total endogenous soluble sugar was measured according to Glassop et al. (2007). Two gram internode tissues of each sample were ground in liquid nitrogen and then extracted with 10 ml deionized water at 70°C. The extractions were centrifuged at 12,000 rpm for 20 min, and the supernatants were collected for the soluble sugar content determination. The sediments were stored at −20°C for starch content determination. The supernatants were freeze-dried and re-dissolved in 1 ml deionized water. A 10 μl aliquot of each sample was injected into an HPLC system (Waters). Glucose, fructose and sucrose were separated on an analytical column (CarboPac PA-100; Dionex Pty Ltd.) and detected by pulsed amperometric detection (Waters).

Starch content was measured according to the method of phenol-sulfuric acid (Dubois et al., 1956). The sediment was dried, weighed and boiled with deionized water. The supernatants were used for the determination of starch content. All mean values were gained by measuring three samples and each sample was measured three times.

### Activities of Sucrose and Starch Metabolizing Enzymes

For the determination of invertase activities, 1 g internode tissues were homogenized in liquid nitrogen and centrifuged in 1 ml extraction buffer containing 50 mM KPO_4_ (pH 7.5), 5 mM MgCl_2_ and 1 mM EDTA by using a refrigerated centrifuge (Xiangyi, Changsha, China) at 12,000 rpm, 4°C for 30 min (Lingle and Dunlap, 1987). The supernatants were collected in dialysis tubing (10 KD cut off, Sigma-Aldrich, Shanghai, China) and dialyzed against 1 l of 10 mM KPO_4_ (pH 7.5) overnight at 4°C. The dialyzate was used for SAI assays. Insoluble extracellular invertase (CWI) is mainly bound to the cell wall, functioning in sucrose unloading (Miller and Chourey, 1992). The pellets were collected and re-suspended in the extraction buffer for the CWI assay.

Activities of SAI and CWI were determined according to Lingle and Dunlap (1987). The extracts were incubated with 1 M sodium acetate buffer (pH 4.5) and 100 mM sucrose at 37°C for 30 min. The reactions were stopped by adding 30 μl of 2.5 M Tris and boiling at 100°C for 5 min. The SAI and CWI activities were expressed in μmol NADH per min per g fresh tissue.

To determine sucrose synthase (SUSY) activities, 1 g tissue samples were homogenized in liquid nitrogen and extracted in 1 ml of extraction buffer containing 50 mM HEPES/NaOH (pH 7.5), 7.5 mM MgCl_2_ and 1 mM EDTA, 2% (w/v) PEG 8000, 2% (w/v) polyvinylpyrrolidone (PVP) and 5 mM dithiothreitol (DTT). The extracts were centrifuged as described above. The supernatant was dialyzed against 1 l of 10 mM HEPES (pH 7.5) overnight at 4°C for SUSY assays (Qazi et al., 2012). The cleavage activities of SUSY were measured according to Lowell et al. (1989). The reaction was preceded at 37°C for 30 min in the extraction buffer containing 80 mM MES buffer (pH 5.5), 5 mM NaF, 100 mM sucrose, and 5 mM UDP. The SUSY activities were expressed in μmol NADH per min per g fresh tissue.

Activities of starch phosphorylase (STP) were determined according to Appeldoorn et al. (1999). One gram fresh tissues were homogenized in liquid nitrogen and incubated in 1 ml extraction buffer containing 100 mM HEPES buffer (pH 7.4), MgCl_2_ 5 mM, EDTA 2 mM, 10% (v/v) glycerol, 0.1% BSA, 5 mM DTT, and 2% (w/v) insoluble PVP, and then centrifuged at 12,000 *g*, 4°C for 30 min. The supernatant was preserved at 4°C. STP activities were determined in a final volume of 1 ml medium containing 50 mM HEPES buffer (pH 7.0), 0.4% soluble starch, 0.4 mM NAD, 1 U phosphoglucomutase, 1 U glucose-6-phosphate dehydrogenase, 15 mM glucose-1,6-bisphosphate and 10 mM Na_3_PO_4_ and 50 μl enzyme extract. The reaction proceeded for 30 min at 37°C. The activities were expressed as μmol NADH per min per g fresh tissue.

### Xylem Transport

Sano et al. (2005) considered that acid fuchsin is diffused more rapidly and more widely than safranin, and its staining is more faithful in tracing water transport. Hence, the acid fuchsin was used in this work to trace the water transport in the bending and normally elongating internodes. The water transport rates in the 8^th^ internode above ground were constantly determined every 3 h by infusing the acid fuchsin solution (4 g l^–1^) into the internodes according to the methods of Sano et al. (2005) and Fritz and Ehwald (2013). The samples were immediately taken back to the lab in a refrigerator (−20°C) and cut into sections for light microscopic observation. The water transport rates were separately determined in the inner and outer sides of the bending internodes.

The *in situ* water potentials of the bending and normally elongating internodes were also constantly determined every hour from 16:00 p.m. to 9:00 a.m. by using PSY1 Stem Psychrometer (ICT International, Armidale, NSW, Australia) at the middle parts of these bending internodes.

The *in situ* water pressure at the middle parts of these internodes was determined by a liquid-pressure transducer (Measurement range: -15 to 50 MPa) (Elecall, Elecall Company, Leqing, China), linked to a syringe needle (0.9 × 28 mm) by a 2 m long PVC pipe (2 mm in diameter). The syringe needle was inserted into the culm walls of the bending internode, and the pipe was filled with water before the water pressure was measured. The *in situ* water pressures were constantly measured every 3 h from 18:00 p.m. to 9:00 a.m. in the inner and outer sides of the bending internodes. These experiments were constantly performed in six young bamboo shoots at the same time for three consecutive days.

### Phloem Transport

In order to trace the assimilate transport rates in the phloem of bending internodes, an aqueous working solution of 5,6-carboxyfluorescein diacetate (CFDA, Sigma-Aldrich, St. Louis, MO, United States) was prepared by diluting a 1% CFDA stock solution (in acetone, stored at −20°C) in 10 mM phosphate-buffered saline (PBS, pH 6.8), and the final concentration was about 30 μg ml^–1^. The introduction of CFDA was conducted according to the methods of Hancock et al. (2008); Milne et al. (2015) and Wang et al. (2020). The phloem transport rates of the inner and outer sides of the bending internodes were separately determined. Before the determination, the waxy cuticle of the 8th internodes of 1-month-old young shoots was scraped. The CFDA solution was painted on the epidermis of the bare sides by using a cotton wool ball. In order to determine the transport rate in the outer sides of the bending internodes, a small hole (about 0.1–0.2 cm in diameter) was made on the shoot sheaths, and the CFDA solution was painted on the epidermis of the outer sides. This experiment was also constantly measured every 3 h from 18:00 p.m. to 9:00 a.m. After the tracing experiment, all the internodes were cut off and stored in a refrigerator (−20°C), then taken back into the lab for imaging under a fluorescence microscope.

### Statistical Analysis

The data in this paper were the means of three independent experiments with 3–5 replicates for each experiment (means ± SD). The significance between means was statistically evaluated with analysis of variance by using SPSS 13.0 software (SPSS, Inc., Chicago, IL, United States), and the least significant difference (LSD) test was employed. Differences were considered significant at *P* < 0.05. The schematic diagram of the physiological mechanism of the internode bending growth was designed using EdrawMax software (Edraw Software Co., Ltd., Shenzhen, China).

## Results

### Bending Growth of Internodes

The excision of shoot sheaths affected the normal elongation of bamboo internodes (Figure 1). When a strip of shoot sheath was peeled off at the position close to the node at morning (9:00 a.m.), the elongating internodes would slightly bend after 10 h (19:00 p.m.) or did not bend any more (Figure 1C). While it was cut in the late afternoon (19:00 p.m.), the internodes would significantly bend toward the bare side at the next morning (9:00 a.m.) (Figure 1F). All the bare internodes were observed to bend at the position close to the nodes, which was usually the elongation zone of the internodes (Figure 1B). According to the continuous observations of the internode bending growth, we noticed that the internodes bent rapidly at midnight from 0:00 to 3:00 a.m. (Figures 1E,H). When a strip of culm sheath was peeled off instead of a black plastic film enclosing the bare internodes, it would still bend during the night. This phenomenon implied that the internode bending growth is unrelated to the changes of light or temperature. As the internodes completed their elongation, the internodes did not curve any more (Figure 1G).

According to the microscopic observations on the longitudinal sections of the bending internodes (middle part, Figure 1B), it was noticed that the parenchyma cells were shorter in the inner sides than the outer sides (Figures 1I,J and Table 1). The metaxylem vessels were also longer in the outer sides than the inner sides (Figures 1K,L and Table 1). Additionally, the parenchyma cells and metaxylem cells were both shorter in the outer sides of the bending internodes than in the normally elongating internodes (control culms), indicating that the excision of shoot sheaths not only affected the elongating growth of the inner sides, but also affected that of the outer sides. However, it did not significantly affect the vascular bundle areas between the inner and outer sides of the bending internodes (Table 1).

**TABLE 1:** Morphological difference of different cells between the inner and outer sides of the curving *Fargesia yunnanensis* internodes.

Position	Parenchyma cells (μm)		Metaxylem vessels (μm)	Vascular bundle area (μm^2^)
		
	Length	Width	Length	
Inner sides	58.56 ± 9.63c	50.51 ± 7.12a	439.95 ± 63.46b	339015.98 ± 22708.21a
Outer sides	102.91 ± 14.80b	51.62 ± 5.75a	732.65 ± 104.95a	360267.95 ± 50371.80a
Control	116.94 ± 11.60a	53.27 ± 9.78a	740.27 ± 122.72a	347899.00 ± 33646.30a

*Note: Different letters indicated significant difference at *P* < 0.05.*

By the stain of Hoechst 33342, we noticed that most of the nuclei of the parenchyma cells in the outer side were close to the walls, while those of the parenchyma cells in the inner side were usually localized in the center of the cells (Figures 1M,N). Under TEM, it was noticed that the vacuolization of parenchyma cells in both the inner and outer sides was high (Figures 1O–R). However, the expansion degrees of vacuoles were higher in the parenchyma cells of the outer sides than those of the inner sides in the bending internodes. The nuclei of parenchyma cells were also smaller and pressed against the walls in the outer sides, and the cytoplasm layers were also thinner in the outer sides than the inner zones (Figures 1O–R).

### Starch Metabolism in the Bending and Normally Elongating Internodes

**FIGURE 2:**
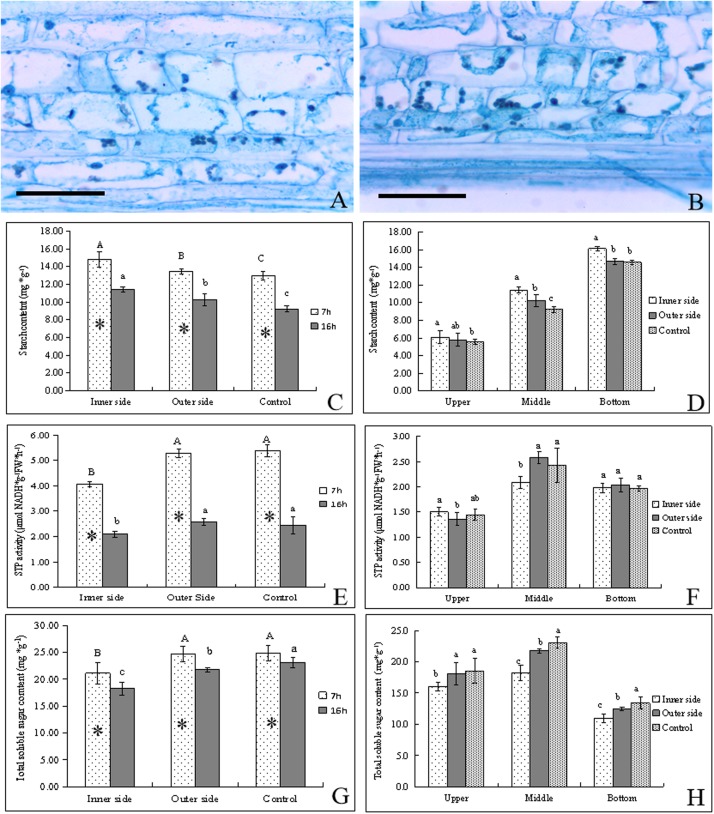
Starch catabolism in the bending and normal elongating internodes. ^∗^ indicated significant difference between different time at *P* < 0.05. Different letters indicated significant difference between different parts of the internodes at *P* < 0.05. **(A)** Starch grains localized in parenchyma cells of the outer side of the bending internodes, showing fewer starch grains. Bar = = 50 μm. **(B)** Starch grains localized in parenchyma cells in the inner side of the bending internodes, showing more starch grains. Bar = 50 μm. **(C)** Starch contents in the inner and outer sides at different time, showing higher starch contents after 7 h at midnight and higher contents in the inner sides. **(D)** Starch distribution in longitudinal directions at 16 h after a strip of culm sheath was peeled off, showing the highest contents in the inner side and the highest contents in the bottom. **(E)** STP activities in the inner and outer sides at different time, showing higher activities at midnight and in the outer side and normally elongating internodes. **(F)** STP activities in longitudinal directions at 16 h after a strip of culm sheath was peeled off. **(G)** Total soluble sugar contents in the inner and outer sides at different time. **(H)** Total soluble sugar contents in longitudinal directions at 16 h after a strip of culm sheath was peeled off.

**FIGURE 3:**
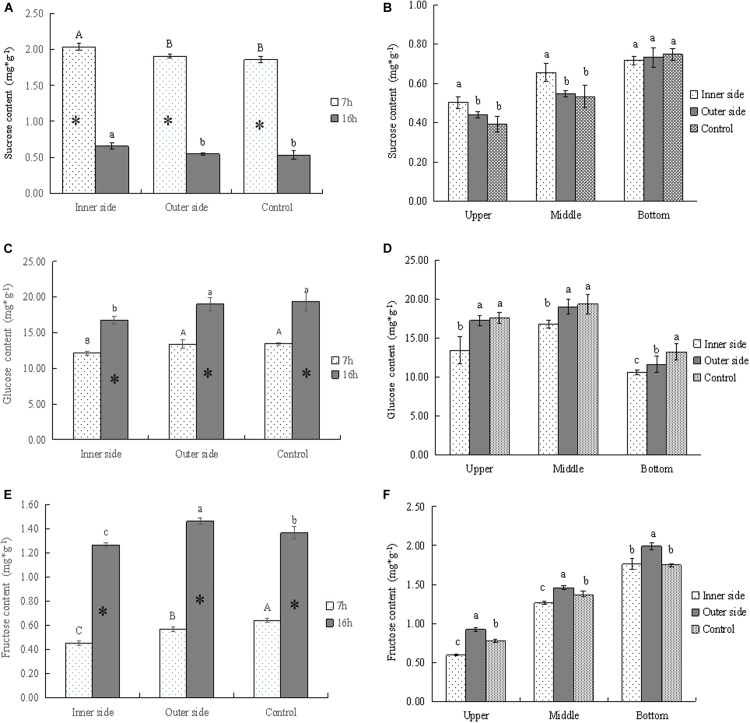
Soluble sugars in the bending and normally elongating internodes. ^∗^ indicated significant difference between different times at *P* < 0.05. Different letters indicated significant difference between different parts of the internodes at *P* < 0.05. **(A)** Sucrose contents in the inner and outer sides of the bending internodes at different times, showing the highest sucrose content in the inner side and higher content after 7 h of the sheath excision. **(B)** Sucrose contents in longitudinal directions at 16 h after a strip of culm sheath was peeled off, showing the highest contents in the bottom of the bending and normally elongating internodes. Higher sucrose contents were also shown in the inner sides of the middle and upper parts of the bending internodes. **(C)** Glucose contents in the inner and outer sides of the bending internodes at different times, showing lower content in the inner sides as compared to that in the outer sides and normally elongating internodes. The glucose content also increased from mid night (after 7 h) to the next morning (after 16 h). **(D)** Glucose contents in longitudinal directions at 16 h after a strip of culm sheath was peeled off, showing higher content in the upper and middle parts. The content was lower in the inner sides of all the parts than in the outer sides and normally elongating internodes. **(E)** Fructose contents in the inner and outer sides of the bending internodes at different times, showing a significant increase of contents with time and lower contents in the inner sides. **(F)** Fructose contents in longitudinal directions at 16 h after a strip of shoot sheath was peeled off, showing the highest contents in the bottom of all internodes and the lowest contents in the inner sides of all the parts.

**FIGURE 4:**
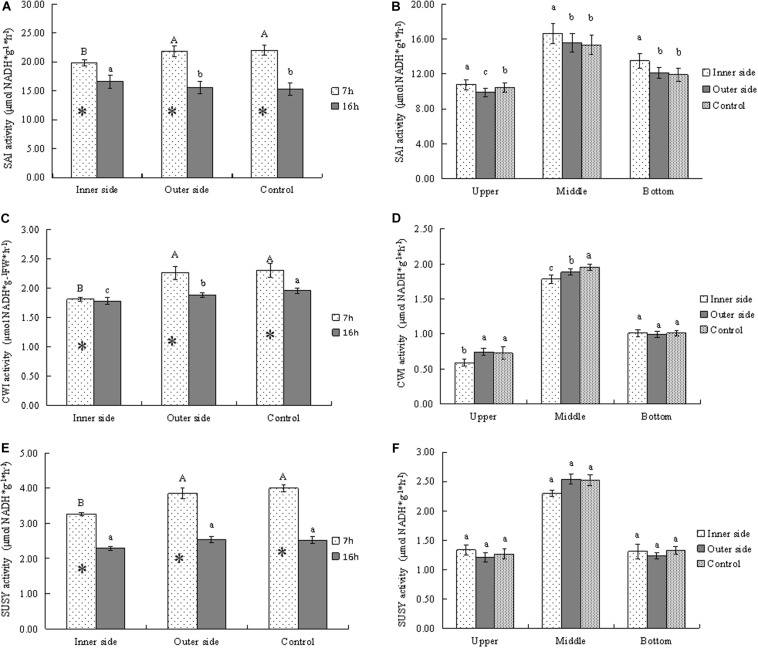
Activities of sucrose metabolizing enzymes in bending and normally elongating internodes. ^∗^ indicated significant difference between different times at *P* < 0.05. Different letters indicated significant difference between different parts of the internodes at *P* < 0.05. **(A)** SAI activities in the inner and outer sides of the bending internodes at different time, showing higher activities after 7 h than after 16 h and lower activities in the inner sides. **(B)** SAI activities in longitudinal directions at 16 h after a strip of culm sheath was peeled off, showing the highest activities in the middle parts. **(C)** Glucose contents in the inner and outer sides of the bending internodes at different time, showing lower activities in the inner sides and higher activities after 7 h than after 16 h. **(D)** Glucose contents in longitudinal directions at 16 h after a strip of culm sheath was peeled off, showing the highest activities in the middle parts. **(E)** Fructose contents in the inner and outer sides of the bending internodes at different time, showing higher activities after 7 h than after 16 h and lower activities in the inner sides. **(F)** Fructose contents in longitudinal directions at 16 h after a strip of culm sheath was peeled off, showing the highest activities and the middle parts of all the internodes.

In order to explore the physiological mechanism of internode bending growth, the differences of sugar metabolism between the inner and outer sides of the bending internodes were analyzed at midnight and the next morning (Figures 2–4). The sugar metabolism was also compared among the upper, middle and bottom parts (Figure 1B) of the bending and the normally elongating internodes after 16 h of the shoot sheath excision at the next morning.

By PAS staining, it was observed that the size and number of starch grains in the parenchyma cells were smaller and fewer in the outer sides than the inner sides in the bending internodes (Figures 2A,B). By determining starch contents, we found that the starch contents were significantly higher in these internodes at midnight (after 7 h) than the next morning (after 16 h) in both the inner and outer sides of the bending internodes (Figure 2C). The normally elongating internodes showed the same trend. This result implied that internode elongation consumed a great deal of starch during one night’s growth, and led to a significant decrease of starch content in internodes. In addition, the starch content was always significantly less in the outer sides than the inner sides, but was higher than that in the normal elongating internodes. This revealed that the outer sides consumed more starch than the inner sides in the bending internodes, but less compared to the normal elongating internodes. The excision of shoot sheaths not only affected the starch consumption of the inner sides of the bending internodes, but also affected that of the outer sides during the internode bending growth. However, the effects of the excision of shoot sheaths on starch consumption were slighter in the outer side than the inner side. The excision of the culm sheath significantly impeded starch degradation in the bare sides of the internodes.

By comparison, it was also observed that the starch contents showed a downward trend in upper < middle < bottom in both the inner and outer sides of the bending internodes and in the normally elongating internodes (Figure 2D). The starch contents were higher in the inner sides than the outer sides at all parts of these internodes. Therefore, the excision of shoot sheaths affected the starch hydrolysis in all parts.

STP activities were higher after 7 h than after 16 h in both the bending internodes and the control, showing higher starch degradation at midnight than the next morning (Figure 2E). Meanwhile, the STP activities were always significantly lower in the inner sides than the outer sides and the control, which implied a significant decrease of starch degradation in the inner sides due to the excision of shoot sheaths. In a comparison of STP activities among different parts of the bending and normally elongating internodes, it was observed that the middle parts had higher STP activities than the upper and bottom parts, which were the bending sites of the internodes and also the elongating zone of the normally elongating internodes (Figure 2F).

Soluble sugar contents in internodes at different times were found to be higher at midnight (after 7 h) than at the next morning (after 16 h) (Figure 2G). The total soluble sugar contents were always lower in the inner sides than the outer sides of the bending internodes and the normally elongating internodes at midnight and the next morning. The middle parts had higher contents of total soluble sugar than other parts in the bending and normally elongating internodes (Figures 2G,H).

Generally, starch degradation was lower in the inner sides as compared to the outer sides, which led to higher starch contents but lower soluble sugar contents in the inner sides. However, the phenomenon of higher STP activities and soluble sugar contents but lower starch contents in the outer sides than the inner sides also revealed a decrease of starch degradation in the inner side of the bending internodes, and the starch degradation was mainly used to increase the soluble sugar concentrations in the elongating zones of the internodes. The excision of shoot sheaths impeded starch hydrolysis and the increase of soluble sugar content in the bare side of the internodes.

### Sucrose Metabolism in the Bending and Normally Elongating Internodes

By determining the concentrations of sucrose, glucose, and fructose in the bending and normally elongating internodes, we noticed that glucose content was far higher than that of other soluble sugars, being very close to the total soluble sugar content (Figures 2G, 3A,C,D). Therefore, glucose was the major constituent of the total soluble sugar in the internodes, which could reach up to 83.42, 81.98, and 81.72% of the total soluble sugar in the inner and outer sides of the bending internodes and the normal elongating internodes at midnight. It could reach up to 91.93, 87.28, and 83.92% in the inner sides, outer sides and the normal elongating internodes at the next morning. The high proportion of glucose in the total soluble sugars indicated that the glucose catabolism did not play a key role in internode elongation, while the degradation of starch and sucrose was more important for increasing the soluble sugar contents in the rapidly elongating internodes. The increase of glucose content from midnight to the next morning showed constant sucrose degradation.

The sucrose contents significantly decreased with time, while glucose and fructose showed significantly higher contents after 16 h, which revealed the constant sucrose catabolism in the bending and normally elongating internodes (Figures 2G, 3A,C,E). Additionally, the sucrose contents were far higher in the inner sides than the outer sides and the normally elongating internodes after 7 and 16 h (Figure 3A), while both of the glucose and fructose contents were significantly lower in the inner sides than the outer sides and the control (Figures 3C,D). This result indicated lower sucrose degradation in the inner side of the bending internodes as compared to the outer sides and the normally elongating internodes.

The upper and middle parts showed higher sucrose contents but lower glucose and fructose contents in the inner sides of the upper and middle parts as compared to those in the outer sides and the normally elongating internodes (Figures 3B,D,F). While in the bottom parts of the bending internodes, the contents of sucrose, glucose and fructose were lower in the inner sides than the outer sides in the bending internodes. These results showed that the excision of shoot sheaths affected the sucrose catabolism of different parts of the internodes in different degrees.

In order to analyze the sucrose catabolism in the bending internodes, the activities of the related enzymes were determined at different time points (Figure 4). SAI activities were higher after 7 h than 16 h in all internodes, which implied higher SAI activities at midnight than at the next morning (Figure 4A). By comparison of SAI activities between the inner and outer sides of the bending internodes, we noticed that the inner sides showed significantly lower SAI activities at midnight but higher activities the next morning, as compared to the outer sides and the control (Figure 4A). This indicated that the SAI activities varied with time, and the sucrose degradation catabolized by SAI was lower in the inner sides than the outer sides and the normally elongating internodes at midnight. Higher SAI activities at morning might be caused by more sucrose but less glucose in the inner sides of the bending internodes the next morning (Figure 3). SAI activities were significantly higher in the middle parts than the top and bottom parts in both the bending and normally elongating internodes the next morning (Figure 4B). The high SAI activities in the middle parts showed that SAI activities were related to internode elongation.

CWI activities were always lower in the inner sides than the outer sides and the control (Figure 4C). CWI activities of the bending and normally elongating internodes were also higher after 7 h than 16 h (Figure 4C), which showed a similar trend to that of SAI activities. Additionally, CWI activities were significantly higher in the middle parts than the top and bottom parts in the bending and normally elongating internodes (Figure 4D).

SUSY activities were also higher after 7 h than after 16 h in both the inner and outer sides of the bending internodes and the normally elongating internodes (Figure 4E). Similarly, the inner sides of the bending internodes always showed lower SUSY activity values as compared to the outer sides and the normally elongating internodes (Figures 4E,F). After 16 h, the SUSY activities were also higher in the middle parts than the upper and bottom parts of the bending and normally elongating internodes (Figure 4F).

Generally, the activities of the starch- and sucrose-metabolizing enzymes were significantly lower in the inner sides than in the outer sides and normally elongating internodes at midnight, completely consistent with the dynamical changes of starch and soluble sugars in the bending and normally elongating internodes. Moreover, the internodes also bended quickly at midnight after the shoot sheaths were stripped. Therefore, the excision of shoot sheaths impeded the starch and sucrose metabolism in the elongating zones (middle parts) of the internodes.

### Water and Assimilate Transport in the Bending and Normally Elongating Internodes

**FIGURE 5:**
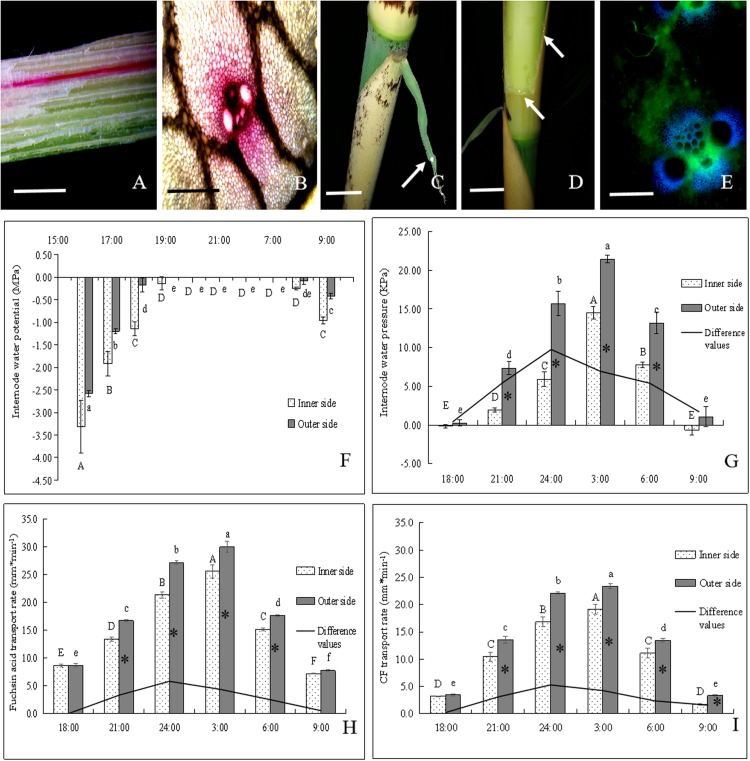
The transportation of water and assimilate in bending internodes, as indicated by fuchsin acid and CF tracers. ^∗^ indicated significant difference between different times at *P* < 0.05. Different letters indicated significant differences between different parts of the internodes at *P* < 0.05. **(A,B)** Fuchsin acid transport in the longitudinal and transverse sections of the curved internodes. **(A)** Bar = 0.5 cm. **(B)** Bar = 200 μm. **(C)** Normally elongating internodes at 0:00 a.m., showing the guttation (white arrow) of shoot sheath blades. Bar = 2.5 cm. **(D)** Water constantly leaked out of the incision of the shoot sheath at night (white arrows). Bar = 2.5 cm. **(E)** CF distribution in vascular bundles of the curved internodes. Bar = 200μm. **(F)** Changes of water potentials in the inner and outer sides of the bending internodes during the period from 16:00 p.m. to 9:00 a.m., showing the significant increase with time until at 19:00 p.m. and then the significant decrease at the next morning (8:00 a.m.). **(G)** Changes of water pressure in the inner and outer sides of the bending internodes during the period from 18:00 p.m. to 9:00 a.m. The water pressure increased significantly with time and reached up to the highest values at 3:00 a.m., and then decreased significantly with time. However, the difference values reached their maximum at 00:00 a.m. and the water pressure values were always significantly higher in the outer sides than the inner sides. **(H)** Fuchsin acid transport rates in the inner and outer sides of the bending internodes during the period from 18:00 p.m. to 9:00 a.m. The water transport rates increased significantly with time and also reached up to the highest values at 3:00 a.m., and then decreased significantly with time. However, the difference values reached their maximum at 00:00 a.m. and the water transport rates were always significantly higher in the outer sides than the inner sides. **(I)** CF transport rates in the inner and outer sides of the bending internodes during the period from 18:00 p.m. to 9:00 a.m., which indicated that the assimilate transport rates increased significantly with time and also reached up to the highest values at 3:00 a.m., and then decreased significantly with time. However, the difference values reached their maximum at 24:00 p.m. and the assimilate transport rates were always significantly higher in the outer sides than the inner sides.

In order to further analyze the physiological mechanism of the internode bending growth, the water and assimilate transport was also determined in the bending and normally elongating internodes by using acid fuchsin and CF (Figures 5A,B,E). It was observed that apparent guttation occurred at midnight (24:00 p.m.) and small water drops constantly accreted on the surface of the sheath blades (Figure 5C). When a strip of sheath was cut off, amounts of water drastically spilled out at the cut surface at midnight (Figure 5D).

**FIGURE 6:**
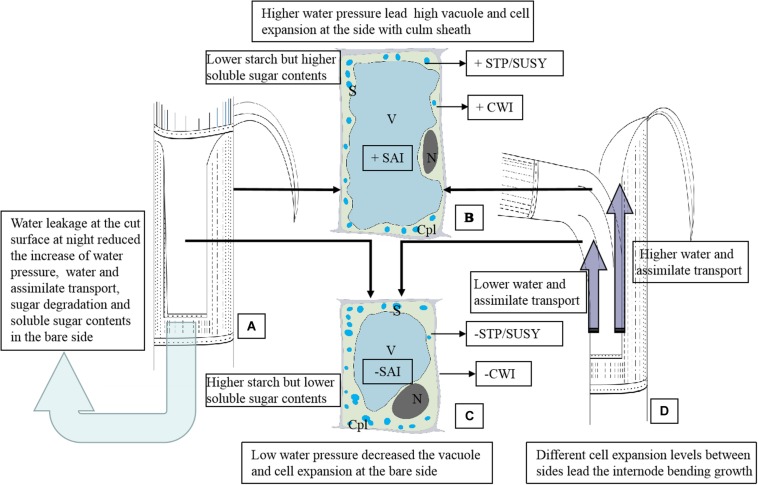
The schematic diagram of the anatomical and physiological mechanism of the internode bending growth of *Fargesia yunnanensis*. SAI, soluble acid invertase. CWI, Insoluble extracellular invertase. SUSY, sucrose synthase. STP, starch phosphorylase. S, starch grains. N, nucleus. Cpl, cytoplasm layer. V, vacuole. **(A)** The shoot internode with a strip of sheath excision. Water leakage constantly occurred at the cut surface during the night, which reduced the increase of water pressure, water and assimilate transport, sugar degradation and soluble sugar contents in the bare side. The light blue arrow indicated the water leakage from the cut of the shoot sheath. **(B)** Parenchyma cells in the outer sides of the bending internodes suffered higher water pressure, showing a bigger cell size with a larger vacuole, fewer starch grains, a smaller nucleus and thinner cytoplasm layer. There is lower starch but higher soluble sugar contents in these parenchyma cells due to higher activities of SAI, CWI, SUSY, and STP. Soluble sugars accumulation in parenchyma cells reduced the water potentials and further promoted water influx for cell enlargement. +CWI indicated higher CWI activities. +SAI indicated higher SAI activities. +STP/SUSY indicated higher activities of STP and SUSY. **(C)** Parenchyma cells in the bare sides of the bending internodes showed a small cell size with a smaller vacuole but a wider cytoplasm layer due to the lower water pressure as compared to those in the outer sides. There is higher starch but lower soluble sugar contents in these parenchyma cells due to lower activities of SAI, CWI, SUSY, and STP. The low water pressures in the bare sides were mainly caused by the water leakage at the cut of the shoot sheaths. -CWI indicated lower CWI activities. -SAI indicated lower SAI activities. -STP/SUSY indicated lower activities of STP and SUSY. **(D)** The bending internodes, showing a higher transport rate of water and assimilates in the outer sides but lower transport rates in the inner sides, which were separately indicated by the long and short dark blue arrows.

The water potentials of both the outer and inner sides increased significantly with time during the night. They reached their highest values (0 MPa) at 8:00 p.m. and then began to decrease at 8:00 a.m. the next morning (Figure 6E). The outer sides always had higher water potentials than the inner sides, and reached their highest water potentials earlier than the inner sides during the internode bending process. This revealed that the outer sides could increase their water potentials more rapidly as compared to the inner sides. The water pressure also significantly increased with time, reached their highest values at 3:00 a.m., and then decreased constantly (Figure 5G). The water pressure values were also always significantly higher in the outer sides than the inner sides. The water transport rates in the inner and outer sides of the bending internodes also showed an increasing trend with time with the highest values at 3:00 a.m., and then decreased constantly (Figure 5H). Similarly, the water transport rates were also significantly higher in the outer sides than the inner sides.

As for the assimilate transport rates, the completely same trend was shown to that of water transport in the bending internodes during the night (Figure 5I). The values were also always significantly higher in the outer sides than the inner sides. Additionally, the differences of water pressure, water transport rates and assimilate transport rates between the inner sides and the outer sides also constantly increased with time, with the highest values at 24:00 p.m. (Figures 5G–I). Therefore, the excision of shoot sheaths led to a significant decrease in water and assimilate transportation in the inner sides of the bending internodes, as compared to that in the outer sides and the normally elongating internodes.

## Discussion

Shoot sheaths play an important role in protecting the tender lower part of bamboo internodes (Wong, 2004). Once the shoot sheaths were stripped from the elongating shoots, the shoots were more easily broken at the bottom parts of the elongating internodes. Therefore, almost all studies were concentrated on the mechanical functions in protecting the tender shoots (Niklas, 1990, 1998; Zebrowski, 1992; Wong, 2004; Singh et al., 2013), while the physiological functions of shoot sheaths on the internode growth of young bamboo shoots were neglected. Our previous studies have reported that the excision of shoot sheaths could affect the height growth of bamboo shoots (Wang et al., 2017). When one part of culm sheaths was longitudinally stripped from the elongating internodes, the internodes bent toward the bare side rapidly in one night. However, systematic work on the physiological mechanism of shoot sheaths affecting the internode elongation is lacking. Here we systematically discuss all data, in order to understand better which are the key factors controlling the bending process.

### Morphological and Anatomical Characteristics of the Bending Internodes

The outer sides of the bending internodes showed longer parenchyma cells and metaxylem vessels as compared to the inner sides. However, there was no significant difference in the area of vascular bundles between the inner and outer sides. Therefore, we considered that the longitudinal excision of culminating sheaths significantly impeded the expansion of parenchyma cells and metaxylem vessels in the bare sides of the elongating internodes, and then the elongating internodes bent toward the bare sides because of the significant difference in cell elongation between the bare sides and the sides covered with shoot sheaths.

Additionally, most parenchyma cells showed their nuclei in the center in the inner sides of the bending internodes, but close to the walls in the outer sides under a fluorescence microscope and TEM. The cytoplasm was also pressed against the cell walls by the turgor pressure from the vacuoles, and a thinner cytoplasm layer was formed in the parenchyma cells of the outer sides of the bending internodes as compared to that in the parenchyma cells of the inner sides. These results indicated that the parenchyma cells in the outer sides of the bending internodes suffered higher turgor pressure from the vacuoles. Vacuoles are a large organelle and occupy a significant volume (up to 90%) in plant cells, and drive cell expansion by building up the turgor pressure (Zhang et al., 2014) and pushing the cytoplasm against the surrounding cell wall, forming a thin layer (Lodish et al., 2000).

The bending growth of the elongating internodes was mainly caused by the expansion differences of the parenchyma cells and metaxylem vessels between the inner and outer sides. However, the expansion difference of different cells may be explained by the difference of turgor pressure coming from vacuoles. Cell expansion requires an increase in the osmotic pressure of vacuoles and cell extension (Yamada et al., 2009a). For cell expansion, the accumulation of osmotica and influx of water are required (Norikoshi et al., 2013). Hence, it is also essential to analyze the differences of tissue water pressures between the inner and outer sides of the bending internodes.

### Water Transport in Bending Internodes

Water potentials increased significantly with time during the night, and then decreased by dawn in the internodes. Water pressure values and transport rates showed a similar trend during the internode bending growth and reached their highest values during the period from 24:00 p.m. to 3:00 a.m. The difference values of water pressure and water transport rates between the inner and outer sides also reached their maximum at 00:00 a.m. Interestingly, the dynamic of internode bending growth was completely in accordance with the trend of the water pressure and water transport rates in the internodes, which also reached their maximum values at the same time. Hence, the internode bending growth was extremely related to the differences of water transport rates and internode water pressure values between the inner and outer sides during the internode bending growth.

Wegner (2013) considered that plant roots could maintain a positive pressure in the xylem to push water upwards and supply the shoots with water to maintain their growth. The turgor pressure primarily determines the corresponding pressure in the vessels (and vice versa) due to the tight hydraulic connection and the water equilibrium between the two compartments (Thürmer et al., 1999). An increase of tissue water pressure with time during the night contributed to the increase of turgor pressure of vacuoles, and then drove the cell and internode expansion. However, the excision of the shoot sheath limited the increase of water pressure in the bare sides, because most water leaked out at the cut of the shoot sheath, which decreased the turgor pressure of vacuoles and further hindered the cell expansion of the bare sides of the internodes, the growth differences between the bare sides and the sides covered with culm sheaths, and finally the bending growth of the internodes.

Moreover, the thinner cytoplasm layers in the parenchyma cells of the outer sides as compared to the inner sides also revealed the differences of turgor pressure from vacuole expansion between the inner and outer sides of the bending internodes. The outer sides of the bending internodes suffered higher turgor pressure as compared to the inner sides because of their thinner cytoplasm layers in parenchyma cells. The water pressures in internodes increased quickly at 00:00 a.m., and the water transport rates and water potentials began to attain their maximum values. This was also the time when the internodes bent quickly and the water was drastically leaking out at the cut surface of the shoot sheaths. It was further confirmed that the internode bending growth was directly correlated with the water leakage from the cut of the shoot sheaths. The water leakage from the cut surface of the shoot sheaths impeded the increase of water pressure and water influx in the cells of the bare sides.

When the internodes completed their elongation growth, the parenchyma cells deposited their secondary walls with cellulose and lignin (Wang et al., 2017), which inhibited their expansion. Cell expansion is regulated not only by cell turgor pressure, but also by cell wall pressure, which is determined by wall strength (Yamada et al., 2009a, b). Therefore, the internodes did not bend any more, once their elongation completed.

### Sucrose Transport and Metabolism in Bending Internodes

The elongating internodes did not bend or slightly bend during the daytime but bent significantly at night after a part of culm sheaths was stripped. This phenomenon implies that water transport driven by daytime transpiration was not the direct factor that lead to the bending growth of the elongating internodes.

During the night, the excision of culm sheath lead to significant differences in sugar transport and metabolism between the bare sides and the sides covered with shoot sheaths. Our previous study has revealed that the assimilate transport was significantly promoted by water transport and water pressure in young bamboo shoot culms at night (Deng et al., 2020). Culm/stem pressure usually promotes the phloem transport (Savage et al., 2016). After the culm sheaths were excised, most water leaked out at the cut surface of the shoot sheaths, which decreased the water transport rates and water pressures in internodes, and further limited the phloem transport in the bare parts of the bending internodes. Therefore, the assimilate transport rates were lower in the bare sides as compared to the sides covered with sheaths, as indicated by CF trace. Soluble sugars as the osmotica in cells could lower the water potentials and promote water influx and then increase the turgor pressure in cells. However, the increase of soluble sugar content was affected not only by starch and sucrose degradation in cells, but also by sucrose influx. Therefore, we employed CF to trace the phloem transport velocities, and compared the difference between the two sides of the culm walls. The results implied that the excision of culm sheaths might limit the sucrose influx in cells by lowering the phloem transport in the bare sides, limiting the increase of soluble sugars in cells, which also further hindered the cell expansion of the bare sides.

Accordingly, the starch and soluble sugar contents as well as the related enzyme activities were affected by the excision of shoot sheaths in the elongating internodes. Higher starch but lower soluble sugar contents and lower STP activities in the bare sides of the bending internodes implied that the starch catabolism was also impeded by the excision of shoot sheaths. Similarly, higher sucrose contents but lower glucose and fructose contents as well as lower activities of SAI, CWI, and SUSY in the bare sides also revealed lower sucrose catabolism levels. In potato, tuber respiration declined rapidly as O_2_ level decreased, which also reduced invertase activities (Herman et al., 2016). Shoot sheaths are the main transpiration and respiration organs of bamboo shoots through a large number of stomata and high activities of transpiration and respiration (Wang et al., 2018). Therefore, the excision of culm sheaths might reduce the oxygen absorption of the bare sides of the internodes, impeding the hydrolysis of starch and sucrose and further decreasing the total soluble sugar contents. Additionally, the proportion of glucose in the total soluble sugars was also higher in the bare sides than in the sides covered with sheaths and the normally elongating internodes, which might imply that the glucose oxidation was inhibited and the aerobic glycolysis of glucose was impeded by the excision of shoot sheaths.

The outer sides of the bending internodes and the normally elongating internodes had higher contents of total soluble sugars, among which the glucose accounted for the biggest proportion. Soluble sugar accumulation in parenchyma cells reduced the water potentials and promoted water influx for cell enlargement (Yamada et al., 2009a). Some enzymes, such as invertase and SUSY, are involved in the accumulation of soluble sugars and then are required for cell expansion (Yamada et al., 2009a). Higher soluble sugar contents acted as osmotica and further promoted the elongation of the outer sides of the bending internodes. Additionally, the highest proportion of glucose in the total soluble sugars implied that the cell expansion and internode elongation mainly relied on the increase of turgor pressure in cells, rather than the consumption of energy produced from the glycolysis of glucose. The cell expansion was mainly caused by the accumulation of soluble sugars and water influx. Wei et al. (2019) reported that sugar plays an important role in promoting bamboo internode elongation. A similar conclusion was also reported by Wang et al. (2020), stating that the starch- and sucrose-metabolizing enzymes correlated well with the bamboo internode elongation.

Generally, the increase of tissue water pressures in the bare sides of the elongating internodes weakened due to the water leakage from the cut surface of the shoot sheaths, which also impeded the cell expansion of the bare sides, and further caused the internode bending growth, while in the sides covered with shoot sheaths and the internodes with intact sheaths, the cells elongated more rapidly. The increase of water pressure and the water influx in cells play a pivotal role in promoting the cell expansion and in the further rapid growth of bamboo shoots at night. This is in agreement with the previous work that bamboos in tropical regions grow more rapidly during the night (Kaushik et al., 2015). Fang et al. (2019) also considered that the nighttime water influx is highly important for the support of the rapid growth of bamboo shoots.

Therefore, shoot sheaths are not only an important organ for the transpiration and respiration of shoots (Wang et al., 2018), but can also regulate the dynamic changes of water pressure and water transport in the attached shoot internodes by guttation during the night and transpiration during the day. As the internodes complete their elongation, the cells deposit their secondary walls, and then the cells and internodes arrest their elongation. The sheaths drop off and no longer function in regulating water transport and pressure besides the leaves. Shoot sheaths play an important role in the rapid growth of bamboo shoots as a controller in water and assimilate transportation.

## Conclusion

In order to better present the anatomical and physiological mechanism of bamboo internode bending growth, we established a schematic diagram (Figure 6). Bamboo internode elongation mainly relies on the increase of water tissue pressure and total soluble sugar concentration. Once the shoot sheaths were cut, most water leaked at the cut of the shoot sheaths (Figure 6A), which impeded the increase of the water pressure, water and assimilate transport rates, and even impeded the starch and soluble sugar hydrolysis, lowering the activities of SAI, CWI SUSY, and STP in the bare side of the internodes (Figure 6C). While in the outer sides of the bending internodes, higher soluble sugar contents further promoted water influx and vacuole expansion (Figure 6B). Thinner cytoplasm layers were observed in the parenchyma cells in the sides covered with shoot sheaths (the outer sides), which also revealed higher water pressure in these cells. The transport rates of water and assimilates were also impeded in the bare sides (Figure 6D).

Higher water influx and sugar transport and catabolism increased the turgor pressure and promoted cell expansion in the outer sides as compared to the bare sides (Figures 6B,C), and then the internodes bent (Figure 6D). The glucose metabolism should not play a key role in cell expansion and internode bending growth, because glucose always occupied the biggest proportion of the total soluble sugars in both the bending internodes and the normally elongating internodes during the night. The bending growth of bamboo internodes was mainly due to the significant differences in cell expansion, which was led by the differences in water pressures and sugar hydrolysis (starch and sucrose) levels in the elongating zones between the inner and outer sides of the internodes. Shoot sheaths played an important role in the rapid growth of bamboo shoots as one controller by regulating water transport and pressure as well as the sugar transport and metabolism in the internodes.

## Data Availability Statement

All datasets generated for this study are included in the article/supplementary material.

## Author Contributions

SW designed the experiments and wrote the manuscript. HZ carried out the water transport experiment. PL and CC carried out the enzyme activities determination and anatomical experiment. JL and CW provided financial support for the experiments and revised the manuscript.

## Conflict of Interest

The authors declare that the research was conducted in the absence of any commercial or financial relationships that could be construed as a potential conflict of interest.
